# Predicting invasive species impacts: a community module functional response approach reveals context dependencies

**DOI:** 10.1111/1365-2656.12292

**Published:** 2014-10-20

**Authors:** Rachel A Paterson, Jaimie T A Dick, Daniel W Pritchard, Marilyn Ennis, Melanie J Hatcher, Alison M Dunn

**Affiliations:** 1 Institute for Global Food Security, School of Biological Sciences, Queen's University BelfastBelfast, UK; 2 School of Biology, University of LeedsLeeds, UK; 3 School of Planning, Architecture and Civil Engineering, Queen's University BelfastBelfast, UK; 4 School of Biological Sciences, University of BristolBristol, UK

**Keywords:** *Gammarus*, indirect effect, non-native, parasitism, predator cue, predator–prey, interaction

## Abstract

1. Predatory functional responses play integral roles in predator–prey dynamics, and their assessment promises greater understanding and prediction of the predatory impacts of invasive species.
2. Other interspecific interactions, however, such as parasitism and higher-order predation, have the potential to modify predator–prey interactions and thus the predictive capability of the comparative functional response approach.
3. We used a four-species community module (higher-order predator; focal native or invasive predators; parasites of focal predators; native prey) to compare the predatory functional responses of native *Gammarus duebeni celticus* and invasive *Gammarus pulex* amphipods towards three invertebrate prey species (*Asellus aquaticus*, *Simulium* spp., *Baetis rhodani*), thus, quantifying the context dependencies of parasitism and a higher-order fish predator on these functional responses.
4. Our functional response experiments demonstrated that the invasive amphipod had a higher predatory impact (lower handling time) on two of three prey species, which reflects patterns of impact observed in the field. The community module also revealed that parasitism had context-dependent influences, for one prey species, with the potential to further reduce the predatory impact of the invasive amphipod or increase the predatory impact of the native amphipod in the presence of a higher-order fish predator.
5. Partial consumption of prey was similar for both predators and occurred increasingly in the order *A. aquaticus*, *Simulium* spp. and *B. rhodani*. This was associated with increasing prey densities, but showed no context dependencies with parasitism or higher-order fish predator.
6. This study supports the applicability of comparative functional responses as a tool to predict and assess invasive species impacts incorporating multiple context dependencies.

## Introduction

The impacts of invasive species are of key socio-economic concern and are widely recognised as major drivers of global biodiversity loss (Salo *et al*. 2007; Crowl *et al*. 2008; Davis 2009; Simberloff *et al*. 2013). In response, invasion ecology research is increasingly focused on developing techniques that can reliably assess and ultimately predict these impacts (e.g. invasion history: Ricciardi 2003; Kulhanek, Ricciardi & Leung 2011a; niche modelling: Kulhanek, Leung & Ricciardi 2011b). Previously, where predatory impacts of invasive species have caused concern, maximum feeding rates have been assessed by providing predators with a single density of prey (e.g. Fielding *et al*. 2003; Renai & Gherardi 2004; Rehage, Barnett & Sih 2005; Stoffels *et al*. 2011). However, such ‘snapshot’ designs largely ignore the population consequences of predation, by obscuring the often nonlinear relationship between prey density and the number of prey killed (i.e. the functional response; Holling 1966).

More recently, a growing body of work has utilised predatory functional responses to explore the impact of invasive predators (Dick *et al*. 2014). Functional responses play an integral role in predator–prey interactions (Jeschke, Kopp & Tollrian 2002) and may provide greater insight into how invasive predators impact prey populations, especially at lower, more ecologically relevant, prey densities (Dick *et al*. 2014). For example, as the density of a focal prey species declines, predators may kill increasingly high proportions of prey and thus might drive prey locally extinct (Type II functional response). Alternatively, prey may exploit a low-density refuge, such as when the predator switches to an alternative prey species (Type III functional response). A large number of mathematical extensions to the basic Type II and Type III responses have been developed (for reviews see Juliano 2001; Jeschke, Kopp & Tollrian 2002; Dick *et al*. 2014), however, the defining characteristics of each remain; a Type II functional response follows a saturating (hyperbolic) curve defined by a constant (density-independent) attack rate (*a*), which controls the initial slope of the curve and the handling time (*h*), which limits the maximum number of prey consumed; whereas in a Type III response, the attack rate (*a*) is itself a function of density, which decreases as prey density reduces, underpinning the characteristic ‘S-shaped’ curve of a Type III functional response.

Whilst functional response techniques have been used extensively in biological control research to assess impacts of control agents (e.g. Madadi *et al*. 2011; Carrillo & Peña 2012; Latham & Mills 2012), this technique has only recently been applied as a comparative tool in invasion biology (Dick *et al*. 2014). There is growing evidence that invasive species frequently exhibit higher functional response curve asymptotes (*i.e*. a lower handling time) than their native counterparts (e.g. Dubs & Corkum 1996; Bollache *et al*. 2008; Haddaway *et al*. 2012; Dick *et al*. 2013). Furthermore, such differences in functional responses of native and invasive predators often reflect changes to invaded community structure (Dick *et al*. 2013), leading to the suggestion that comparison of functional responses could be a useful approach to predict and assess invasive species impacts (Dick *et al*. 2014).

Functional responses are often regarded as the gold standard for quantitative measurement of predatory interactions, but classical functional response approaches neglect the community context in which such interactions take place. To date, comparative functional response approaches have mostly considered predatory impacts on a single prey species (e.g. Bollache *et al*. 2008; Dick *et al*. 2010; Haddaway *et al*. 2012); however, predatory functional responses can differ with prey type (e.g. Elliott 2003). Field observations also demonstrate that the severity of invasive species predatory impacts may vary among prey species (e.g. Matsuzaki *et al*. 2009), thus leading Dick *et al*. (2014) to highlight the importance of assessing functional responses across a wide variety of prey species. Furthermore, predator–prey dynamics in natural communities may be influenced by other context dependencies, such as higher-order predators (i.e. predators that consume other predators) that exhibit trait-mediated (nonconsumptive) effects on intermediate predator and/or prey behaviour (Ohgushi, Schmitz & Holt 2012; Alexander, Dick & O'Connor 2013a; Barrios-O'Neill *et al*. 2014). Parasite infections in predators and/or prey may also modify the outcome of predator–prey interactions (Fenton & Rands 2006; Hatcher, Dick & Dunn 2006) and may increase (acanthocephalan infected *Gammarus pulex* (L.) amphipods, Dick *et al*. 2010) or decrease (microsporidian infected white-clawed crayfish *Austropotamobius pallipes*, Haddaway *et al*. 2012), the functional responses of parasitised hosts. Thus, integrating context dependencies into experimental comparative functional response approaches may strengthen their utility in understanding and predicting invasive species impacts.

In this study, we take a novel approach of using a four-species community module of closely interacting species (Holt 1997; Gilman *et al*. 2010) to assess the predatory functional responses of invasive vs. native intermediate predators. Such an approach bridges the gap between the artificially simplistic dynamics of one- or two-species interactions and the often mechanistically intractable complexity of whole ecosystem experiments. Our community module, consisting of a higher-order predator, focal native or invasive predator, parasites of focal predators and native prey species, allows us to examine how processes, such as predation and parasitism, simultaneously interact to influence native or invasive predator–prey dynamics. This approach potentially strengthens the utility of predatory functional responses in invasion ecology contexts.

In Ireland, the invasive predatory amphipod *G. pulex* has replaced the native Irish congener *Gammarus duebeni celticus* Stock & Pinkster in many rivers and lakes (Dick, Montgomery & Elwood 1993; MacNeil *et al*. 2001, 2004). This invasive amphipod has caused widespread reductions in invertebrate community abundance and diversity (e.g. *Baetis rhodani*, *Simulium* spp., Kelly *et al*. 2006) and has been observed to partially consume invertebrate prey (Ennis, *pers. comm*.). Both amphipod species are prey for fish including brown trout and are also host to parasites that may alter their predatory impact (Fielding *et al*. 2003; MacNeil *et al*. 2003b; Dick *et al*. 2010). The trophically transmitted fish acanthocephalan parasite, *Echinorhynchus truttae* Schrank, infects both amphipod species (prevalence – *G. pulex*: 0–70%, *G. d. celticus*: 0–1%, MacNeil *et al*. 2003b,c). *Pleistophora mulleri* (Pfeiffer; Georgevitch), a microsporidian parasite transmitted horizontally by contact among individual amphipods, infects the native amphipod only (prevalence 0–90%, MacNeil *et al*. 2003a). In our study, we utilise the comparative functional response approach to simultaneously measure the predatory impact of native and invasive amphipods on three key native prey species and to assess how parasitism and the presence of a higher-order predator influences these interactions.

## Materials and methods

### Study Organisms

The community module consisted of focal native or invasive predators, parasites of focal predators, native prey and higher-order predator. Focal amphipod predators; Male *G. d. celticus* (mean length ± SD [pereon and pleosome, Gledhill, Sutcliffe & Williams 1993]: 10·7 ± 1·2 mm) were collected by kick-net from the Downhill stream, County Antrim (55·166674N, 6·8201185W), and male *G. pulex* (mean length ± SD: 10·0 ± 1·3 mm) were obtained from the Minnowburn, County Down, Northern Ireland (54·548509N, 5·9526063W). Female amphipods were not used in our experiments as their predatory ability may vary with the presence of offspring in their brood pouch. Parasitism; Parasite status of each amphipod was initially determined by the presence of an *E. truttae* cystacanth (in *G. pulex*) or *P. mulleri* spore mass (in *G. d. celticus*) clearly visible through the host exoskeleton and was confirmed after the experiment by dissection. Prey; Three native invertebrate species (*Asellus aquaticus* (L.) isopods, *Simulium* spp. dipteran larvae, *Baetis rhodani* Pictet ephemeropteran nymphs) were selected as prey for the functional response experiments. These species vary in terms of their relative mobility (*Simulium* spp. < *A.  aquaticus* < *B. rhodani*), exoskeleton robustness (*A. aquaticus* > *B. rhodani* > *Simulium* spp.) and represent the macroinvertebrate communities that are negatively impacted by *G. pulex* invasion (Kelly *et al*. 2003, 2006). Although all prey species were present at both amphipod collection sites (Paterson pers. obs.), for ease of collection, prey were obtained from sites where they were locally abundant (*Simulium* spp.: length 5–6 mm, Dunore stream, County Antrim 54·680729N, 6·2251382W; *A. aquaticus*: 5–7 mm, Clandeboye Estate, County Down 54·641068N, 5·7139969W; *B. rhodani*: 10–12 mm, Downhill stream). All invertebrates were housed separately, by species and parasitism status (amphipods only), in aquaria containing aerated stream water, substrate and leaf material from their source prior to the experiment. Higher-order fish predator; Commercially raised brown trout *Salmo trutta* L. (fork length 110–130 mm) were maintained in aquaria of aerated filtered stream water on a diet of commercial fish pellets *ad libitum*. All animals were housed in controlled climate facilities (12-h day/12-h night period, 12°C) prior to and during experiments.

### Experimental Design

For each of the three prey species (*A. aquaticus*, *Simulium* spp., *B. rhodani*), we employed a randomised, balanced, fully factorial design, with four treatments: focal amphipod predator (two levels: *G. d. celticus* or *G. pulex*), parasitism (two levels: unparasitised or parasitised), higher-order fish predator (two levels: present or absent) and prey density (seven densities: 2, 4, 6, 8, 10, 20, 30 individual prey). Each treatment combination was replicated four times, resulting in a total of 224 experiments per prey species. Due to seasonal availability of prey and laboratory space constraints, experiments were carried out sequentially over a period of 6–8 weeks for each prey species (*B. rhodani*, *A. aquaticus*, *Simulium* spp.), with 22 experimental replicates initiated every three days. Adult amphipod predators were acclimatised in the laboratory for 7 days and experiments undertaken a controlled laboratory environment to minimise any potential effects of the time when the experiment was undertaken. Amphipods were held individually without food for 48-h in filtered stream water (container dimensions: diameter 60 mm, volume 80 ml) prior to the experiment to standardise hunger levels. Fish were randomly assigned to individual experimental aquaria (260 × 210 × 180 mm, semi-opaque plastic, 15 cm between aquaria) containing 5 L of continuously aerated filtered stream water and held without food for 24-h. Individual amphipods were assigned to separate experimental glass pots (diameter 90 mm, height 50 mm) containing 150 ml of filtered stream water and prey of a given species and density (*n* = 4 replicates per density). Experimental pots were covered with fine gauze mesh and placed in experimental aquaria (one pot per aquaria with or without fish), thus exposing both the amphipod and their prey to the visual and olfactory cues of the higher-order predator, but preventing the consumption of invertebrate prey by this higher predator. Previous work by Andersson *et al*. (1986) suggests *G. pulex* does not respond to the visual cues of a fish predator if olfactory cues are absent. During the 40-h experimental period, consumed prey were not replaced. For each community module, control pots (*n* = 4 replicates per prey density) were also set up to measure the survival of each prey species in the absence of the amphipod predator and presence/absence of the higher-order fish predator. Thus, we ascertained whether prey death was solely attributable to predation by amphipods or was affected by higher-order predator cues, as predator cue is known to strongly influence invertebrate behaviour (see Paterson *et al*. 2013).

At the end of the experimental period, the total number of prey killed and the number of partially eaten prey (when two or more prey were killed and partially consumed) were recorded. Amphipods were frequently observed to be consuming prey at the end of the experimental period; thus, the presence of single partially consumed prey in aquaria was not considered as evidence of partial prey consumption. Prey mortality (in the absence of amphipod predators) was assessed from each control pot in terms of the number of prey dead (including any cannibalised individuals, *A. aquaticus* only). Amphipods were euthanised by immersion in carbonated water, prior to confirmation of parasitism status by dissection. Replicates in which amphipods moulted during the experimental period were excluded from further analysis and repeated with another randomly selected amphipod (*n* = 10–16), as moulting adversely affects *Gammarus* feeding behaviour (Hargeby & Petersen 1988). Similarly, replicates where *E. truttae*-infected *G. pulex* harboured early development stage *E. truttae* acanthella (pre-infective juvenile worm with adult structures absent) and/or multiple cystacanths (infective juvenile stage with adult structures developed) were also excluded and repeated (*n* = 8–16/prey species), as acanthocephalan age and infection intensity may influence amphipod response to predator cues (Franceschi *et al*. 2008; Dianne *et al*. 2011).

### Statistical Analysis

All statistical analyses were performed using R v. 3.0.3 (R Core Team 2014). To account for the potential bias caused by variation in experimental day between replicates, we graphically examined the relationship between day and the number of prey killed within each community module, with plots indicating the absence of bias. Functional response methods described here are available in an integrated package for functional response analysis in R (*frair*, Pritchard 2014;). Extensions to the methods used by frair are detailed in full in Appendix S1 (Supporting information).

### Phenomenological Functional Response Analysis

To establish whether the relationship between prey density and the number of prey killed is best described by a Type II or a Type III response, a phenomenological approach focusing on the overall shape of the response curve was used (see Jeschke, Kopp & Tollrian 2002; Alexander, Dick & O'Connor 2013b). For each experimental combination, logistic regressions of proportion of prey killed (encompassing both partially and completely eaten prey) against prey density were performed (frair::frair_test). Type II functional responses were indicated by a significant negative first-order term, whereas a significant positive first-order term followed by significant negative second-order term indicated a Type III functional response (Juliano 2001; Pritchard 2014). When results from logistic regressions were inconclusive, we compared Type II and III functional response models with a linear (Type I) functional response (Holling 1966) using Akaike Information Criterion (AIC).

### Mechanistic Functional Response Analysis

Where analyses indicated Type II functional responses were most appropriate, we fitted the random predator equation (eqn 1, Rogers 1972), which accounts for prey depletion and their non-replacement over time;

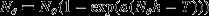
eqn 1where *N*_*e*_ is the number of prey eaten, *N*_*0*_ is the initial prey density, *T* is the total time available and *a* and *h* are the mechanistically interpretable coefficients for attack rate and handling time, respectively. This equation is solved using the Lambert *W* equation (eqn 2, emdbook::lambertW, version 1.3.4, Bolker 2013), parameterised in the following form:

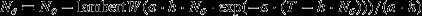
eqn 2

To determine the effect of different treatment combinations on the attack rate (*a*) and handling time (*h*), we used an ‘indicator variable’ approach (Juliano 2001) to model these differences explicitly. Briefly, this approach substitutes the parameters of interest (i.e. *a* and *h*) with terms including that parameter plus a predictor coded to the treatment of interest (an ‘indicator’). In this study, we used so-called treatment coding to compare each treatment level against a base (intercept) value. For example, using this approach (eqns 3–4), the attack rate (*a*) or handling time (*h*) modified by an effect for prey item becomes:

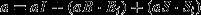
eqn 3

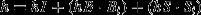
eqn 4where *aI* and *hI* are the attack rate and handling time for the base (intercept) level of the treatment (in this case *A. aquaticus*), *aB* and *hB* are the difference in *a* and *h*, respectively, from this base level attributable to the *B. rhodani* treatment, *aS* and *hS* are the difference attributable to the *Simulium* spp. treatment and *B*_*i*_ and *S*_*i*_ are indicator variables coded as 1 for *B. rhodani* or *Simulium* spp. treatments, respectively, and zero otherwise. For testing within each community module, this approach was extended to higher-order interactions terms using methods generalised from standard linear regression, using an additive interaction term (Appendix S1, Supporting information). Standard regression outputs (effect estimates, standard errors, *z*-scores, *P*-values) were used to construct relevant contrasts between groups and to establish if the fitted *a*, *h* and treatment coefficients (*aB*, *aS*, *hB*, *hS* etc.) were significantly different from zero.

Functional response models were fitted using maximum likelihood estimation (bbmle::mle2, version 1.0.52, Bolker and R Development Core Team 2014). We first fitted a single model with indicator variables for the three community modules (*A. aquaticus*, *B. rhodani*, *Simulium* spp.) to test for differences between the modules (eqns 3 and 4), then fitted one model per module to assess the effect of amphipod species, fish presence and parasitism (four models in total, see Appendix S1, Supporting information). Preliminary analysis and visual inspection of the data indicated that attack rate did not differ significantly within the community modules (Table S2, Supporting information); therefore, we fitted a simplified model with no treatment effects (amphipod species, fish presence or parasitism) for attack rate. To visualise the uncertainty around the fitted functional responses, bootstrapping (*n* = 1500) was used to construct empirical 95% confidence intervals of the fitted functional responses.

### Partial Prey Consumption

The effects of amphipod species, parasitism, higher-order fish predator and prey density on the partial consumption of prey were assessed with nonparametric Kruskal–Wallis (K–W) tests. A nonparametric test was used since data violated assumptions of normality and heterogeneous variances implicit with parametric linear modelling approaches.

## Results

Prey survival was high in the absence of amphipods (>96·5%); thus, the majority of prey mortality in the experimental treatments could be ascribed to amphipod predation.

### Phenomenological Functional Responses

Logistic regressions indicated that 22 out of 24 of the four-species community modules (higher-order fish predator; focal native or invasive predators; parasites of focal predators; native prey) displayed Type II functional responses (Table S1, Figs S1–S3, Supporting information). For the two exceptions (*P. mulleri* parasitised *G. d. celticus* consuming *A. aquaticus* in the absence of fish, Appendix S1, Fig. S1d, Supporting information; and unparasitised *G. pulex* consuming *B. rhodani* in the presence of fish, Fig. S3e, Supporting information), comparison of AIC values with a Type I (i.e. a linear) response curve indicated that Type II functional responses were more appropriate fits for these community modules (AIC Type I vs. Type II: 112·5 vs. 102·6; 109·6 vs. 100·0).

### Mechanistic Functional Responses

**Figure 1:**
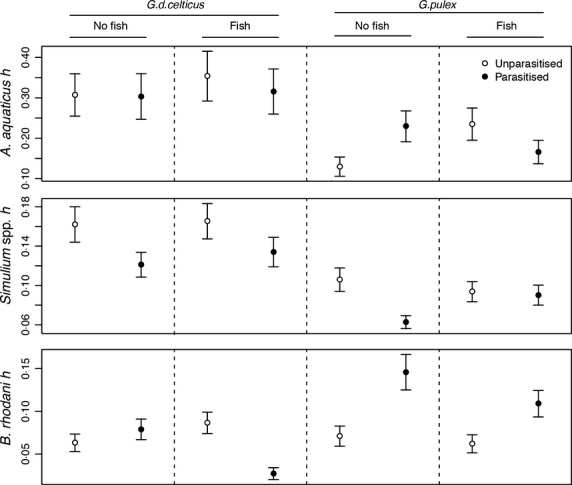
Interactions between parasitism and amphipod species or higher-order fish predator on the handling time (*h*) for *Asellus aquaticus*, *Simulium* spp. and *Baetis rhodani* prey. Circles indicate the mean and error bars are standard error, *n* = 56 per marker).

Overall, functional response attack rates were highest in *Simulium* spp. prey modules (*P *<* *0·001) and did not differ between *A. aquaticus* or *B. rhodani* prey (*P *>* *0·05; Table1). Handling time differed significantly between all prey modules (all *P *<* *0·001), with *A. aquaticus* modules showing highest handling times, whereas handling times were lowest with *B. rhodani* prey (Fig.1).

**Table 1:** Between community module (higher–order fish predator, focal amphipod predator, parasitism) differences in functional response attack rates (*a*) and handling times (*h*) of *Asellus aquaticus*, *Simulium* spp. and *Baetis rhodani* prey. Parameter estimates calculated using the ‘indicator variable’ approach (Juliano 2001; Appendix S1, Supporting information), with statistically significant parameters (α = 0.05) in bold

Base prey species	Parameter	Contrast	Estimate	SE	z value	*P* (z)
*A. aquaticus*	*a*	**Intercept**	0·992	0·155	6·399	**<0·001**
		*B. rhodani*	0·257	0·183	1·403	0·161
		***Simulium*** **spp**.	1·317	0·260	5·078	**<0·001**
	*h*	**Intercept**	0·238	0·0207	11·510	**<0·001**
		***B. rhodani***	−0·167	0·0216	−7·747	**<0·001**
		***Simulium*** **spp.**	−0·131	0·022	−6·102	**<0·001**
*B. rhodani*	*a*	**Intercept**	1·246	0·097	12·870	**<0·001**
		*A. aquaticus*	−0·241	0·185	−1·305	0·192
		***Simulium*** **spp.**	1·131	0·240	4·708	**<0·001**
	*h*	**Intercept**	0·071	0·006	11·860	**<0·001**
		***A. aquaticus***	0·170	0·022	7·856	**<0·001**
		***Simulium*** **spp.**	0·037	0·008	4·400	**<0·001**

**Figure 2:**
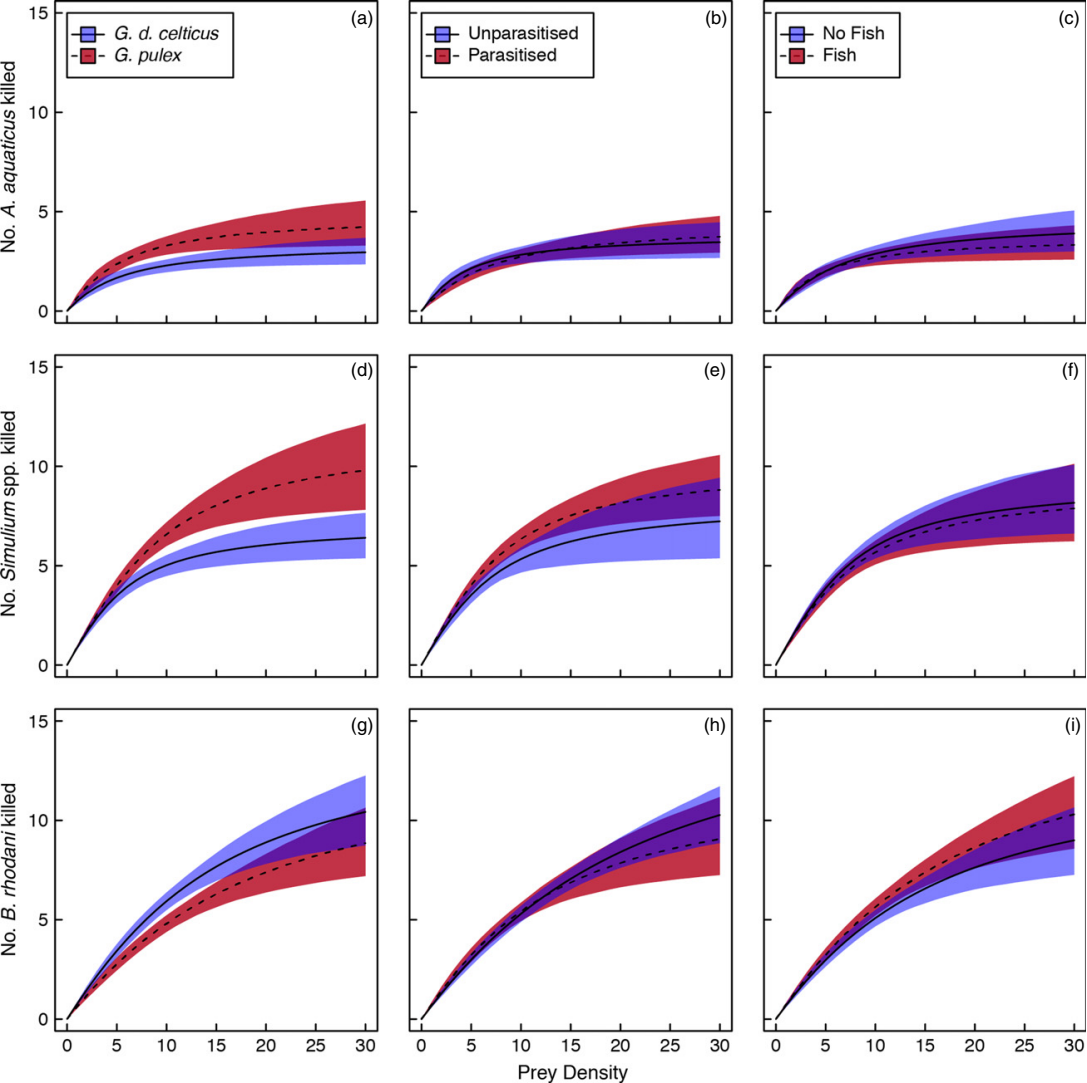
The effects of amphipod species, parasitism and higher-order fish predator on the functional response towards *Asellus aquaticus* (a–c), *Simulium* spp. (d–f) and *Baetis rhodani* (g–i) prey. Lines indicate mean functional response, and coloured bars are 95% equi-tailed confidence intervals.

Handling times of invasive *G. pulex* towards *A. aquaticus* and *Simulium* spp. prey were lower than that of native *G. d. celticus* (all *P *<* *0·001, Table2). This was reflected in the higher functional response curve asymptotes for the invasive species, though some overlap in the 95% confidence intervals were also observed at low and high prey densities (Fig.2a,d). Handling times of amphipod predators to *A. aquaticus* and *Simulium* spp. prey were not influenced by parasitism, fish presence or their higher-order interactions with amphipod species (all *P *>* *0·05, Table2, Fig.1), with highly overlapping functional responses noted within each treatment factor (Fig.2b,c,e,f).

**Table 2:** Within community module differences (higher-order fish predator, focal amphipod predator, parasitism) in functional response attack rates (*a –* intercept only) and handling times (*h*) for *Asellus aquaticus*, *Simulium* spp. and *Baetis rhodani* prey. Parameter estimates calculated using the ‘indicator variable’ approach (Juliano 2001, Appendix S1, Supporting information), with statistically significant differences (α = 0.05) in bold. Base level for each analysis: Native *Gammarus duebeni celticus* – no parasite – no fish

Prey Species	Parameter	Contrast	Estimate	SE	*z* value	*P* (*z*)
*A. aquaticus*	*a*	**Intercept**	0·981	0·142	6·928	**<0·001**
	*h*	**Intercept**	0·307	0·052	5·862	**<0·001**
		**Amphipod (*****G. pulex*****)**	−0·178	0·054	−3·267	**0·001**
		Fish	0·047	0·078	0·596	0·552
		Parasitism	−0·004	0·073	−0·054	0·957
		Amphipod × Fish	0·059	0·089	0·657	0·511
		Amphipod × Parasitism	0·104	0·084	1·237	0·216
		Parasitism × Fish	−0·034	0·108	−0·316	0·752
		Amphipod × Parasitism × Fish	−0·135	0·125	−1·08	0·280
*Simulium* spp.	*a*	**Intercept**	2·564	0·247	10·390	**<0·001**
	*h*	**Intercept**	0·162	0·018	8·988	**<0·001**
		**Amphipod (*****G. pulex*****)**	−0·056	0·020	−2·805	**0·005**
		Fish	0·003	0·024	0·136	0·892
		Parasitism	−0·041	0·021	−1·945	0·052
		Amphipod × Fish	−0·016	0·028	−0·552	0·581
		Amphipod × Parasitism	−0·002	0·024	−0·089	0·929
		Parasitism × Fish	0·010	0·031	0·311	0·756
		Amphipod × Parasitism × Fish	0·030	0·035	0·846	0·397
*B. rhodani*	*a*	**Intercept**	1·362	0·119	11·480	**<0·001**
	*h*	**Intercept**	0·063	0·010	6·190	**<0·001**
		Amphipod (*G. pulex*)	0·008	0·014	0·570	0·569
		Fish	0·023	0·015	1·592	0·112
		Parasitism	0·016	0·014	1·116	0·264
		Amphipod × Fish	−0·032	0·020	−1·611	0·107
		**Amphipod × Parasitism**	0·059	0·026	2·243	**0·025**
		**Parasitism × Fish**	−0·075	0·019	−3·947	**<0·001**
		Amphipod × Parasitism × Fish	0·047	0·034	1·393	0·164

In contrast, handling times towards *B. rhodani* prey were not influenced by the main treatment effects of amphipod species, parasitism or fish presence (all *P *>* *0·05, Table2). However, a significant handling time interaction between amphipod species and parasitism (*P *=* *0·025, Table2) indicated that parasitism by *E. truttae* increased the handling time of *G. pulex* towards *B. rhodani* prey (Figs1 and 2e) more than would have been expected from the additive effects of either parasitism, or invasive amphipod alone. An interaction between parasitism and the higher-order fish predator (*P *<* *0·001) indicated that the presence of both of these factors together caused a reversal in the direction of the additive effect of parasitism or higher fish predator alone (both small increases in handling time, Fig.1), resulting in parasitised *G. d. celticus* individuals exposed to a higher-order fish predators having the lowest handling time of *B. rhodani* prey (Table2, Fig.1).

### Partial Consumption

**Figure 3:**
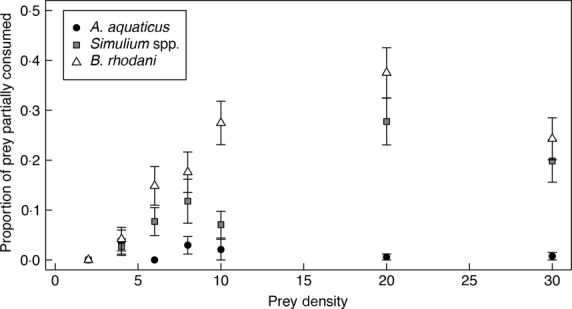
Mean proportion of partially consumed *Asellus aquaticus*, *Simulium* spp. and *Baetis rhodani* prey with increasing density (pooled for amphipod species). Error bars indicate standard error, *n* = 21–32 depending on the occurrence of partial consumption).

The proportion of prey that was partially consumed differed among prey species (Fig.3), with amphipods rarely partially consuming *A. aquaticus* (1%) in comparison to *B. rhodani* (24%) and *Simulium* spp. (14%). Partial consumption was associated with increasing density of *B. rhodani* and *Simulium* spp. prey (K–W density; *H*_7_* *= 113·70, *P *<* *0·001; *H*_7_ = 59·52, *P *<* *0·001, respectively). Amphipod species, parasitism or the presence of the higher-order fish predator did not influence partial consumption of prey (all *P *>* *0·05).

## Discussion

By bridging the gap between artificially simplistic one- or two-species dynamics and the intractable complexity of ecosystems, community modules of three or more interacting species provide a powerful tool to advance our understanding of how processes, such as predation and parasitism, may influence community composition (Holt 1997; Gilman *et al*. 2010). Moreover, as invasive species increasingly become influential components of ecosystems, community modules offer an insight into the processes shaping interactions between invasive species and their recipient communities (Gilman *et al*. 2010). Our use of a four-species community module (higher-order fish predator; focal native or invasive amphipod predators; parasites of focal predators; native prey) revealed key differences in the native and invasive predator functional responses towards different prey species, which can be used to interpret impacts observed in the field. The study also reveals some context dependencies in species interactions within the community modules, emphasising the potential strength for the method in understanding and predicting invasive species impacts (Dick *et al*. 2014).

This study revealed that the overall magnitude of functional responses differed among community modules at the prey species level, as indicated by strong differences in both the attack rate and handling time coefficients. Whilst we are unable to account for potential collinearity between prey species and experimental period, our use of study organism acclimatisation periods and controlled climate facilities, coupled with independence between study replicates, reduces such potential bias and we are confident that the differences observed reflect predator responses to the different prey species. Our study also revealed that differences in functional responses within community modules were driven largely by handling time only. Patterns observed in this study, namely a generally higher *per capita* consumption of prey by invasive *G. pulex,* reflect field observations of community impact (Kelly *et al*. 2003, 2006). Hence, our study gives weight to the proposal that comparative functional response approach could be used to accurately assess invasive species impacts (Dick *et al*. 2014). Recently, MacNeil *et al*. (2013) have confirmed similar congruity between experimental functional responses and field invertebrate patterns, observing that higher functional responses of *G. pulex* than *G. d. celticus* towards invasive *Crangonyx pseudogracilis* prey mirrored field predator–prey associations. That the relative functional response of the native and invasive predator in our study depended on the prey species in question supports suggestions that native and invasive species are rarely functionally redundant, hence invasions, even those involving apparently ‘equivalent’ species, may alter community composition.

Functional responses of predatory amphipods were largely robust to the indirect influences of parasitism and the higher-order fish predator. Whilst a number of previous studies have demonstrated the potential for parasites to either increase (Dick *et al*. 2010) or decrease (Bayoumy 2011; Haddaway *et al*. 2012) the predatory impacts of their hosts, in our study, parasitism alone did not alter the functional responses of parasitised amphipods. Similarly, the presence of fish predator cue has been shown to consistently invoke anti-predator behavioural responses in freshwater invertebrates (Paterson *et al*. 2013); however, such effects may not extend to alterations in the functional responses of predatory invertebrates. Whilst this is somewhat surprising, our results are in line with previous research suggesting that fish predation threats modify *G. pulex* drift activity but not feeding behaviour (Allan & Malmgvist 1989).

The community module approach, however, revealed that predatory functional responses may be influenced by context dependencies involving both host–parasite and parasite–fish interactions. For example, parasitism decreased the functional response of *G. pulex* towards *B. rhodani* prey, whereas parasitism had little effect on *G. d. celticus* predation rates towards the same prey species. Functional responses towards other prey species also indicate that predatory impacts were robust to the influence of parasitism. This, coupled with the observed interaction between parasitism and higher-order fish predators, which indicated that parasitised amphipods in the absence of fish had higher handling times towards *B. rhodani* prey, highlights that in some community modules, the trait-mediated effects of parasitism (i.e. manipulation of host predation behaviour) may only manifest in conjunction with other processes.

For each community module combination, the relationship between prey density and the number of prey killed was best described by a Type II functional response, whereby predators kill increasingly high proportions of prey as prey density declines. No evidence was found to suggest amphipods exhibited Type III functional responses on prey populations. This strongly suggests that predatory amphipods have the potential to drive changes in invertebrate communities as observed from the field (e.g. Kelly *et al*. 2006). However, the non-replacement design experiment employed in our study may not fully encompass the complexity of field predator–prey interactions whereby killed prey may be replaced from a larger prey population source. Therefore, careful consideration is required when designing such experiments to reach an acceptable balance between practicability and the high number of treatment combinations and replication to ensure realistic predator–prey dynamics is captured.

Our study revealed how prey species- and density-dependent partial consumption alters the shape of functional responses and may facilitate greater predatory impacts on prey populations. For instance, we observed that higher functional response asymptotes are associated with the greater degree of partial prey consumption (Figs2 and 3). Furthermore, partial consumption was highest for *B. rhodani* (24%) and extremely rare for *A. aquaticus* (1%, Fig.3). The relative frequency of partial consumption is itself likely to be prey density-dependent, because at high prey densities, predators may feed with increasing selectivity on preferred body parts (as observed in *Macrolophus pygmaeus* (Hemiptera), *Macrobiotus richtersi* (Tardigrada), Jeschke & Hohberg 2008; Fantinou *et al*. 2009). We observed similar behaviour by amphipods feeding on *B. rhodani*, which preferentially consumed the thorax. The incidence of partial consumption of highly active prey, such as *B. rhodani*, may also be explained by prey interference (Mori & Chant 1966; Sandness & McMurtry 1970), since active prey may unintentionally encounter and interrupt the feeding behaviour of a predator. Hammill *et al*. (2010) proposed that prey density-dependent changes in handling time, whereby the handling time per individual prey decreases with increasing prey density, may shift functional responses away from Type II. However, current Type II functional response models assume that handling time is constant with increasing prey density. Further work is therefore required to establish whether current functional response techniques are adequate to deal with such density-dependent changes in predator behaviour, since handling time will limit the shape of functional response curves to a lesser extent. Development of appropriate methodologies to account for partial consumption is a pressing area for future research, particularly given its frequency in certain systems.

In summary, our study indicated that the predatory functional responses of native and invasive amphipods were affected by the species of invertebrate prey, and that trait-mediated effects of parasitism and higher-order fish predators were highly context-dependent. Furthermore, our study highlighted how partial consumption may have prey-specific influences on predatory functional responses. These results support the recommendation by Dick *et al*. (2014) that incorporating multiple prey species is necessary to advance our ability to utilise functional responses in forecasting invasive species impacts. However, further scaling up of comparative functional response experiments may be required to ascertain whether the functional response of an invasive species towards a given prey is conserved in the presence of alternative prey species (but see Smout *et al*. 2010). Recent efforts focusing on predator density-dependent predation have also identified that functional responses may differ between single- and multi-predator experiments (McCoy & Bolker 2008; Barrios-O'Neill *et al*. 2014; Medoc, Spataro & Arditi 2013), with de Villemereuil & López-Sepulcre (2011) proposing that intraspecific, as opposed to interspecific, competition may have greater influences on functional responses. By conducting functional response experiments that reflect realistic biotic communities, we can identify dominant processes that underlie predator–prey dynamics in the environment. Such an approach promises to serve as a powerful tool to predict and assess invasive species impacts.

## Data accessibility

Data are available from the Dryad Digital Repository: http://dx.doi.org/10.5061/dryad.1k894 (Paterson *et al*. 2014). Data package title: Data from: Predicting invasive species impacts: a community module functional response approach reveals context dependencies Data files: Paterson_Functional_Responses

## Supporting Information

**Appendix S1.** Detailed description of functional response model fitting and assessment of between and within community module differences. **Table S1.** Parameter estimates (and significance levels) for logistic regression analyses of proportion of prey killed in relation of initial prey density for each combination of prey species – amphipod species (GDC – *Gammarus duebeni celticus*, GP – *Gammarus pulex*) – parasitism – higher-order (fish) predator **Table S2.** Within community module differences (higher-order fish predator, focal amphipod predator, parasitism) in functional response attack rates (*a*) and handling times (*h*) for *Asellus aquaticus*, *Simulium* spp. and *Baetis rhodani* prey **Fig. S1.** The combined effects of parasitism and higher-order predator on the predatory functional responses of native *Gammarus duebeni celticus* (unparasitised or parasitised with *Pleistophora mulleri*) and invasive *Gammarus pulex* amphipods (unparasitised or parasitised with *Echinorhynchus truttae*) towards *Asellus aquaticus* prey. **Fig. S2.** The combined effects of parasitism and higher-order predator on the predatory functional responses of native *Gammarus duebeni celticus* (unparasitised or parasitised with *Pleistophora mulleri*) and invasive *Gammarus pulex* amphipods (unparasitised or parasitised with *Echinorhynchus truttae*) towards *Simulium* spp. prey. **Fig. S3.** The combined effects of parasitism and higher-order predator on the predatory functional responses of native *Gammarus duebeni celticus* (unparasitised or parasitised with *Pleistophora mulleri*) and invasive *Gammarus pulex* amphipods (unparasitised or parasitised with *Echinorhynchus truttae*) towards *Baetis rhodani* prey.

## References

[b1] Alexander ME, Dick JTA, O'Connor NE (2013a). Trait-mediated indirect interactions in a marine intertidal system as quantified by functional responses. Oikos.

[b2] Alexander ME, Dick JTA, O'Connor NE (2013b). Born to kill: predatory functional responses of the littoral amphipod *Echinogammarus marinus* Leach throughout its life history. Journal of Experimental Marine Biology and Ecology.

[b3] Allan J, Malmgvist B (1989). Diel activity of *Gammarus pulex* (Crustacea) in a south Swedish stream: comparison of drift catches vs. baited traps. Hydrobiologia.

[b4] Andersson KG, Brönmark C, Herrmann J, Malmqvist B, Otto C, Sjörström P (1986). Presence of sculpins (*Cottus gobio*) reduces drift and activity of *Gammarus pulex* (Amphipoda). Hydrobiologia.

[b5] Barrios-O'Neill D, Dick JTA, Emmerson MC, Ricciardi A, MacIsaac HJ, Alexander ME (2014). Fortune favours the bold: a higher predator reduces the impact of a native but not an invasive intermediate predator. Journal of Animal Ecology.

[b6] Bayoumy MH (2011). Foraging behavior of the Coccinellid *Nephus includens* (Coleoptera: Coccinellidae) in response to *Aphis gossypii* (Hemiptera: Aphididae) with particular emphasis on larval parasitism. Environmental Entomology.

[b7] Bolker BM (2013).

[b500] Bolker BM, R Development Core Team (2014). http://CRAN.R-project.org/package=bbmle.

[b8] Bollache L, Dick JTA, Farnsworth KD, Montgomery WI (2008). Comparison of the functional responses of invasive and native amphipods. Biology Letters.

[b9] Carrillo D, Peña JE (2012). Prey-stage preferences and functional and numerical responses of *Amblyseius largoensis* (Acari: Phytoseiidae) to *Raoiella indica* (Acari: Tenuipalpidae). Experimental and Applied Acarology.

[b10] Crowl TA, Crist TO, Parmenter RR, Belovsky G, Lugo AE (2008). The spread of invasive species and infectious disease as drivers of ecosystem change. Frontiers in Ecology and the Environment.

[b11] Davis MA (2009). Invasion Biology.

[b12] Dianne L, Perrot-Minnot MJ, Bauer A, Gaillard M, Leger E, Rigaud T (2011). Protection first then facilitation: a manipulative parasite modulates the vulnerability to predation of its intermediate host according to its own developmental stage. Evolution.

[b13] Dick JTA, Montgomery I, Elwood RW (1993). Replacement of the indigenous amphipod *Gammarus duebeni celticus* by the introduced *Gammarus pulex* - differential cannibalism and mutual predation. Journal of Animal Ecology.

[b14] Dick JTA, Armstrong M, Clarke HC, Farnsworth KD, Hatcher MJ, Ennis M (2010). Parasitism may enhance rather than reduce the predatory impact of an invader. Biology Letters.

[b15] Dick JTA, Gallagher K, Avlijas S, Clarke H, Lewis S, Leung S (2013). Ecological impacts of an invasive predator explained and predicted by comparative functional responses. Biological Invasions.

[b16] Dick JTA, Alexander ME, Jeschke JM, Ricciardi A, MacIsaac HJ, Robinson TB (2014). Advancing impact prediction and hypothesis testing in invasion ecology using a comparative functional response approach. Biological Invasions.

[b17] Dubs DOL, Corkum LD (1996). Behavioral interactions between round gobies (*Neogobius melanostomus*) and mottled sculpins (*Cottus bairdi*. Journal of Great Lakes Research.

[b18] Elliott JM (2003). A comparative study of the functional response of four species of carnivorous stoneflies. Freshwater Biology.

[b19] Fantinou AA, Perdikis DC, Labropoulos PD, Maselou DA (2009). Preference and consumption of *Macrolophus pygmaeus* preying on mixed instar assemblages of *Myzus persicae*. Biological Control.

[b20] Fenton A, Rands SA (2006). The impact of parasite manipulation and predator foraging behavior on predator-prey communities. Ecology.

[b21] Fielding NJ, MacNeil C, Dick JT, Elwood RW, Riddell GE, Dunn AM (2003). Effects of the acanthocephalan parasite *Echinorhynchus truttae* on the feeding ecology of *Gammarus pulex* (Crustacea: Amphipoda). Journal of Zoology.

[b23] Franceschi N, Bauer A, Bollache L, Rigaud T (2008). The effects of parasite age and intensity on variability in acanthocephalan-induced behavioural manipulation. International Journal for Parasitology.

[b24] Gilman SE, Urban MC, Tewksbury J, Gilchrist GW, Holt RD (2010). A framework for community interactions under climate change. Trends in Ecology and Evolution.

[b25] Gledhill T, Sutcliffe DW, Williams WD (1993). British Freshwater Crustacea Malacostraca: A Key with Ecological Notes.

[b26] Haddaway NR, Wilcox RH, Heptonstall REA, Griffiths HM, Mortimer RJG, Christmas M (2012). Predatory functional response and prey choice identify predation: differences between native/invasive and parasitised/unparasitised crayfish. PLoS ONE.

[b27] Hammill E, Petchey OL, Anholt BR (2010). Predator functional response changed by induced defenses in prey. The American Naturalist.

[b28] Hargeby A, Petersen RC (1988). Effects of low pH and humus on the survivorship, growth and feeding of *Gammarus pulex* (L) (Amphipoda). Freshwater Biology.

[b29] Hatcher MJ, Dick JTA, Dunn AM (2006). How parasites affect interactions between competitors and predators. Ecology Letters.

[b30] Holling CS (1966). The functional response of invertebrate predators to prey density. Memoirs of the Entomological Society of Canada.

[b31] Holt RD, Gange AC, Brown VK (1997). Community modules. Multitrophic Interactions in Terrestrial Systems.

[b34] Jeschke JM, Hohberg K (2008). Predicting and testing functional responses: an example from a tardigrade–nematode system. Basic and Applied Ecology.

[b35] Jeschke JM, Kopp M, Tollrian R (2002). Predator functional responses: discriminating between handling and digesting prey. Ecological Monographs.

[b36] Juliano SA, Scheiner SM, Gurevitch J (2001). Nonlinear curve fitting: predation and functional response curves. Design and Analysis of Ecological Experiments.

[b37] Kelly DW, Dick JTA, Montgomery WI, MacNeil C (2003). Differences in composition of macroinvertebrate communities with invasive and native *Gammarus* spp. Freshwater Biology.

[b38] Kelly DW, Bailey RJ, MacNeil C, Dick JTA, McDonald RA (2006). Invasion by the amphipod *Gammarus pulex* alters community composition of native freshwater macroinvertebrates. Diversity and Distributions.

[b39] Kulhanek SA, Leung B, Ricciardi A (2011b). Using ecological niche models to predict the abundance and impact of invasive species: application to the common carp. Ecological Applications.

[b40] Kulhanek SA, Ricciardi A, Leung B (2011a). Is invasion history a useful tool for predicting the impacts of the world's worst aquatic invasive species?. Ecological Applications.

[b41] Latham DR, Mills NJ (2012). Host instar preference and functional response of *Aphidius transcaspicus*, a parasitoid of mealy aphids (Hyalopterus species). BioControl.

[b42] MacNeil C, Montgomery WI, Dick JTA, Elwood RW (2001). Factors influencing the distribution of native and introduced *Gammarus* spp. in Irish river systems. Archiv Fur Hydrobiologie.

[b43] MacNeil C, Dick JTA, Hatcher MJ, Terry RS, Smith JE, Dunn AM (2003a). Parasite-mediated predation between native and invasive amphipods. Proceedings of the Royal Society B: Biological Sciences.

[b44] MacNeil C, Fielding NJ, Dick JTA, Briffa M, Prenter J, Hatcher MJ (2003b). An acanthocephalan parasite mediates intraguild predation between invasive and native freshwater amphipods (Crustacea). Freshwater Biology.

[b45] MacNeil C, Fielding NJ, Hume KD, Dick JTA, Elwood RW, Hatcher MJ (2003c). Parasite altered micro-distribution of *Gammarus pulex* (Crustacea: Amphipoda). International Journal for Parasitology.

[b46] MacNeil C, Prenter J, Briffa M, Fielding NJ, Dick JTA, Riddell GE (2004). The replacement of a native freshwater amphipod by an invader: roles for environmental degradation and intraguild predation. Canadian Journal of Fisheries and Aquatic Sciences.

[b47] MacNeil C, Dick JTA, Alexander ME, Dodd JA, Ricciardi A (2013). Predators vs. alien: differential biotic resistance to an invasive species by two resident predators. NioBiota.

[b48] Madadi H, Parizi EM, Allahyari H, Enkegaard A (2011). Assessment of the biological control capability of *Hippodamia variegata* (Col.: Coccinellidae) using functional response experiments. Journal of Pest Science.

[b49] Matsuzaki S-I, Usio N, Takamura N, Washitani I (2009). Contrasting impacts of invasive engineers on freshwater ecosystems: an experiment and meta-analysis. Oecologia.

[b50] McCoy MW, Bolker BM (2008). Trait-mediated interactions: influence of prey size, density and experience. Journal of Animal Ecology.

[b51] Medoc V, Spataro T, Arditi R (2013). Prey: predator ratio dependence in the functional response of a freshwater amphipod. Freshwater Biology.

[b52] Mori H, Chant DA (1966). The influence of prey density, relative humidity, and starvation on the predacious behavior of *Phytoseiulus persimilis* Athias-Henriot (Acarina: Phytoseiidae). Canadian Journal of Zoology.

[b53] Ohgushi T, Schmitz OJ, Holt RD (2012). Trait-Mediated Indirect Interactions: Ecological and Evolutionary Perspectives.

[b54] Paterson RA, Pritchard DW, Dick JTA, Alexander ME, Hatcher MJ, Dunn AM (2013). Predator cue studies reveal strong trait-mediated effects in communities despite variation in experimental designs. Animal Behaviour.

[b55] Paterson RA, Dick JTA, Pritchard DW, Ennis M, Hatcher MJ, Dunn AM (2014). http://dx.doi.org/10.5061/dryad.1k894.

[b56] Pritchard DW (2014). http://CRAN.R-project.org/package=frair.

[b57] R Core Team (2014). R: A Language and Environment for Statistical Computing.

[b58] Rehage J, Barnett B, Sih A (2005). Foraging behaviour and invasiveness: do invasive *Gambusia* exhibit higher feeding rates and broader diets than their noninvasive relatives?. Ecology of Freshwater Fish.

[b59] Renai B, Gherardi F (2004). Predatory efficiency of crayfish: comparison between indigenous and non-indigenous species. Biological Invasions.

[b60] Ricciardi A (2003). Predicting the impacts of an introduced species from its invasion history: an empirical approach applied to zebra mussel invasions. Freshwater Biology.

[b61] Rogers D (1972). Random search and insect population models. Journal of Animal Ecology.

[b62] Salo P, Korpimäki E, Banks PB, Nordström M, Dickman CR (2007). Alien predators are more dangerous than native predators to prey populations. Proceedings of the Royal Society B: Biological Sciences.

[b63] Sandness J, McMurtry J (1970). Functional response of three species of Phytoseiidae (Acarina) to prey density. The Canadian Entomologist.

[b64] Simberloff D, Martin J-L, Genovesi P, Maris V, Wardle DA, Aronson J (2013). Impacts of biological invasions: what's what and the way forward. Trends in Ecology and Evolution.

[b65] Smout S, Asseburg C, Matthiopoulos J, Fernandez C, Redpath S, Thirgood S (2010). The functional response of a generalist predator. PLoS ONE.

[b66] Stoffels BEMW, Tummers JS, Van der Velde G, Platvoet D, Hendriks HWM, Leuven RSEW (2011). Assessment of predatory ability of native and non-native freshwater gammaridean species: a rapid test with water fleas as prey. Current Zoology.

[b67] de Villemereuil PB, López-Sepulcre A (2011). Consumer functional responses under intra- and inter-specific interference competition. Ecological Modelling.

