# Supplementary material for: Predicting invasive species impacts: a community module functional response approach reveals context dependencies
Source: J Anim Ecol. 2014 Oct 20;84(2):453–63. doi: 10.1111/1365-2656.12292 (PMC4354255; doi:10.1111/1365-2656.12292)
Supplement: Supplementary file 1 — Appendix S1. Detailed description of functional response model fitting and assessment of between and within community module differences. Table S1. Parameter estimates (and significance levels) for logistic regression analyses of proportion of prey killed in relation of initial prey density for each combination of prey species – amphipod species (GDC – Gammarus duebeni celticus, GP – Gammarus pulex) – parasitism – higher-order (fish) predator Table S2. Within community module differences (higher-order fish predator, focal amphipod predator, parasitism) in functional response attack rates (a) and handling times (h) for Asellus aquaticus, Simulium spp. and Baetis rhodani prey Fig. S1. The combined effects of parasitism and higher-order predator on the predatory functional responses of native Gammarus duebeni celticus (unparasitised or parasitised with Pleistophora mulleri) and invasive Gammarus pulex amphipods (unparasitised or parasitised with Echinorhynchus truttae) towards Asellus aquaticus prey. Fig. S2. The combined effects of parasitism and higher-order predator on the predatory functional responses of native Gammarus duebeni celticus (unparasitised or parasitised with Pleistophora mulleri) and invasive Gammarus pulex amphipods (unparasitised or parasitised with Echinorhynchus truttae) towards Simulium spp. prey. Fig. S3. The combined effects of parasitism and higher-order predator on the predatory functional responses of native Gammarus duebeni celticus (unparasitised or parasitised with Pleistophora mulleri) and invasive Gammarus pulex amphipods (unparasitised or parasitised with Echinorhynchus truttae) towards Baetis rhodani prey. [file jane0084-0453-sd1.docx]

**Supporting Information**

**Supplementary Methods**

The approach used here follows the ‘indicator variable’ approach described by Juliano (2001). These supplementary methods describe, in full, the formulae used for fitting the functional response models and extraction treatment-level coefficients. These analyses were performed with R (v. 3.0.3, R Core Team 2014) and the bbmle (v. 1.0.16, Bolker *et al*. 2014) and emdbook (v. 1.3.4, Bolker 2013) packages.

Assessing differences between community modules

The differences in attack rate (*a*) and handling time (*h*) in each community module containing either *Asellus aquaticus* (A), *Simulium* spp. (S) or *Baetis* *rhodani* (B) prey were assessed by fitting the Rogers Type II functional response model to the entire dataset, implemented within R as follows:

$\boldsymbol{N}_{\boldsymbol{e}}\mathbf{=}\boldsymbol{N}_{\boldsymbol{O}}\mathbf{-lambert}\boldsymbol{W}\mathbf{(}\boldsymbol{a}\boldsymbol{\cdot}\boldsymbol{h}\boldsymbol{\cdot}\boldsymbol{N}_{\boldsymbol{O}}\boldsymbol{\cdot exp(-}\boldsymbol{a}\boldsymbol{\cdot(}\boldsymbol{T}\mathbf{-}\boldsymbol{h}\boldsymbol{\cdot}\boldsymbol{N}_{\boldsymbol{O}}\mathbf{)))/(}\boldsymbol{a}\boldsymbol{\cdot}\boldsymbol{h}\mathbf{)}$ Eqn. S1

where *N_e_* is the number of prey eaten, *N_0_* is the initial prey density, *T* is the total time available and *a* and *h* are the attack rate and handling time, and are defined as:

$a=aI+(aB\cdot B_{i})+(aS\cdot S_{i})$ Eqn. S2

$h=hI+(hB\cdot B_{i})+(hS\cdot S_{i})$ Eqn. S3

where *aI* and *hI* are the attack rate and handling time for the base (intercept) level of the treatment (in this case *A. aquaticus*), *aB* and *hB* are the difference in *a* and *h*, respectively, from this base level attributable to the *B. rhodani* treatment, *aS* and *hS* are the difference attributable to the *Simulium* spp. treatment and *B_i_* and *S_i_* are indicator variables coded as 1 for *B. rhodani* or *Simulium* spp. treatments, respectively, and zero otherwise.

To extract the coefficients and relevant contrasts for *A. aquaticus*, the model was refit with *B. rhodani* as the base (intercept) level and *a* and *h* defined as:

$a=aI+(aA\cdot A_{i})+(aS\cdot S_{i})$ Eqn. S4

$h=hI+(hA\cdot A_{i})+(hS\cdot S_{i})$ Eqn. S5

where *aA*, *hA* and *A_i_* coefficients and predictors for *A. aquaticus*, with the same meaning as those defined for *B. rhodani* and *Simulium* spp. prey items (Eqns. S2 and S3), above.

Assessing differences within community models

After assessing differences between the community modules, the effects of amphipod species (S), parasitism (P) and the presence of fish (F) were assessed within each module (*A. aquaticus*, *Simulium* spp. and *B. rhodani* prey) by fitting the Rogers Type II curve (Eqn. S1) with *a* and *h* defined as follows:

$$a=aI+\left( aS\cdot S_{i} \right)+\left( aF\cdot F_{i} \right)+\left( aP\cdot P_{i} \right)+\left( aSF\cdot S_{i}\cdot F_{i} \right)+\left( aSP\cdot S_{i}\cdot F_{i} \right)+\left( aFP\cdot F_{i}\cdot P_{i} \right)+(aSFP\cdot S_{i}\cdot F_{i}\cdot P_{i})$$

Eqn. S6

$$h=hI+\left( hS\cdot S_{i} \right)+\left( hF\cdot F_{i} \right)+\left( hP\cdot P_{i} \right)+\left( hSF\cdot S_{i}\cdot F_{i} \right)+\left( hSP\cdot S_{i}\cdot F_{i} \right)+\left( hFP\cdot F_{i}\cdot P_{i} \right)+(hSFP\cdot S_{i}\cdot F_{i}\cdot P_{i})$$

Eqn. S7

where *aI* and *hI* are the attack rate and handling time for the base (intercept) level of the treatment (*Gammarus duebeni celticus*, fish absent, unparasitised)*, aS*, *aF*, *aP* or *hS*, *hF*, *hP* are the variation in *a* or *h* due to amphipod species, fish presence or parasitism, *aSF*, *aSP*, *aFP* or *hSF*, *hSP*, *hFP* are second-order interaction terms for *a* or *h* due to a combined effect of species-fish (*SF*), species-parasitism (*SP*) or fish-parasitism (*FP*), *aSFP* or *hSFP* are the third-order interaction terms for *a* or *h* due to the combined species-fish-parasitism (*SFP*) effect and *S_i_*, *F_i_* and *P_i_* are the indictor variables coded as either 0 for the base level or 1 for the contrast (*i.e.* *G. d. celticus*, fish present, parasitised).

Following preliminary analysis, which showed that there was no significant effect of amphipod species, parasitism, or the presence of fish on the attack rate (*a*), a reduced model excluding these effects was used. The formulae for *N_e_* (Eqn. S1) and *h* (Eqn. S7) were as above, but a single coefficient for *a* was used. Thus Eqn. S6 was simplified to:

$a=aI$ Eqn. S8

where *aI* is as defined above. No refitting of these models with modified base levels was needed in because every treatment had only two levels and thus all required information was obtained from a single model.

**References**

Bolker B. and R Development Core Team (2014). bbmle: Tools for general maximum likelihood estimation. R package version 1.0.16. <http://CRAN.R-project.org/package=bbmle>

Bolker, B. (2013). emdbook: Ecological Models and Data in R. R package version 1.3.4.

R Core Team (2014). R: A language and environment for statistical computing. R Foundation for Statistical Computing, Vienna, Austria. <http://www.R-project.org/>.

**Table S1.** Parameter estimates (and significance levels) for logistic regression analyses of proportion of prey killed in relation of initial prey density for each combination of prey species – amphipod species (GDC – *Gammarus duebeni celticus*, GP – *Gammarus pulex*) – parasitism – higher-order (fish) predator.

| Prey species | Amphipod species | Parasite | Fish | Type II |  |
| --- | --- | --- | --- | --- | --- |
|  |  |  |  | *N_O_* termCoefficient | *p* |
| *Asellus aquaticus* | **GDC** | **No** | **No** | -0.064 | **<0.001** |
|  | **GDC** | **No** | **Yes** | -0.089 | **<0.001** |
|  | GDC | Yes | No | -0.027 | 0.059 |
|  | **GDC** | **Yes** | **Yes** | -0.076 | **<0.001** |
|  | **GP** | **No** | **No** | -0.059 | **<0.001** |
|  | **GP** | **No** | **Yes** | -0.063 | **<0.001** |
|  | **GP** | **Yes** | **No** | -0.087 | **<0.001** |
|  | **GP** | **Yes** | **Yes** | -0.060 | **<0.001** |
|  |  |  |  |  |  |
| *Simulium* spp. | **GDC** | **No** | **No** | -0.062 | **<0.001** |
|  | **GDC** | **No** | **Yes** | -0.095 | **<0.001** |
|  | **GDC** | **Yes** | **No** | -0.118 | **<0.001** |
|  | **GDC** | **Yes** | **Yes** | -0.074 | **<0.001** |
|  | **GP** | **No** | **No** | -0.059 | **<0.001** |
|  | **GP** | **No** | **Yes** | -0.082 | **<0.001** |
|  | **GP** | **Yes** | **No** | -0.122 | **<0.001** |
|  | **GP** | **Yes** | **Yes** | -0.056 | **<0.001** |
|  |  |  |  |  |  |
| *Baetis rhodani* | **GDC** | **No** | **No** | -0.043 | **<0.001** |
|  | **GDC** | **No** | **Yes** | -0.074 | **<0.001** |
|  | **GDC** | **Yes** | **No** | -0.053 | **<0.001** |
|  | **GDC** | **Yes** | **Yes** | -0.041 | **<0.001** |
|  | **GP** | **No** | **No** | -0.024 | **0.039** |
|  | GP | No | Yes | -0.018 | 0.107 |
|  | **GP** | **Yes** | **No** | -0.057 | **<0.001** |
|  | **GP** | **Yes** | **Yes** | -0.069 | **<0.001** |

**Table S2.** Within community module differences (higher –order fish predator, focal amphipod predator, parasitism) in functional response attack rates (*a*) and handling times (*h*) for *Asellus aquaticus*, *Simulium* spp. and *Baetis rhodani* prey. Parameter estimates calculated using the ‘indicator variable’ approach (Juliano 2001, Supplementary Methods). Base level for each analysis: Native *Gammarus duebeni celticus* – no parasite – no fish.

| Prey species | Parameter | Contrast | Estimate | SE | z value | *p* (z) |
| --- | --- | --- | --- | --- | --- | --- |
| *Asellus aquaticus* | *a* | Intercept | 1.029 | 0.537 | 1.914 | 0.056 |
|  |  | Amphipod | 0.387 | 0.810 | 0.478 | 0.633 |
|  |  | Parasitism | -0.698 | 0.547 | -1.275 | 0.202 |
|  |  | Fish | 0.534 | 0.896 | 0.596 | 0.551 |
|  |  | Amphipod x Parasitism | 0.883 | 1.040 | 0.849 | 0.396 |
|  |  | Amphipod x Fish | -0.797 | 1.190 | -0.670 | 0.503 |
|  |  | Parasitism x Fish | -0.234 | 0.935 | -0.250 | 0.803 |
|  |  | Amphipod x Parasitism x Fish | 0.138 | 1.485 | 0.093 | 0.926 |
|  | *h* | **Intercept** | 0.330 | 0.078 | 4.237 | **<0.001** |
|  |  | Amphipod | -0.170 | 0.088 | -1.946 | 0.052 |
|  |  | **Parasitism** | -0.203 | 0.104 | -1.956 | **0.050** |
|  |  | Fish | 0.0431 | 0.101 | 0.428 | 0.669 |
|  |  | Amphipod x Parasitism | 0.315 | 0.121 | 2.612 | **0.009** |
|  |  | Amphipod x Fish | 0.060 | 0.123 | 0.490 | 0.624 |
|  |  | Parasitism x Fish | 0.090 | 0.140 | 0.645 | 0.519 |
|  |  | Amphipod x Parasitism x Fish | -0.274 | 0.170 | -1.609 | 0.108 |
| *Simulium* spp. | *a* | Intercept | 2.114 | 1.236 | 1.71 | 0.087 |
|  |  | Amphipod | 0.131 | 1.460 | 0.090 | 0.929 |
|  |  | Parasitism | 1.105 | 1.444 | 0.765 | 0.444 |
|  |  | Fish | 1.198 | 1.760 | 0.681 | 0.496 |
|  |  | Amphipod x Parasitism | 0.433 | 1.781 | 0.243 | 0.808 |
|  |  | Amphipod x Fish | -0.757 | 2.054 | -0.369 | 0.713 |
|  |  | Parasitism x Fish | -2.692 | 1.953 | -1.378 | 0.168 |
|  |  | Amphipod x Parasitism x Fish | 0.573 | 2.388 | 0.240 | 0.810 |
|  | *h* | Intercept | 0.162 | 0.044 | 3.658 | **<0.001** |
|  |  | Amphipod | -0.050 | 0.050 | -0.986 | 0.324 |
|  |  | Parasitism | -0.039 | 0.046 | -0.837 | 0.403 |
|  |  | Fish | 0.022 | 0.051 | 0.430 | 0.667 |
|  |  | Amphipod x Parasitism | 0.0009 | 0.052 | 0.017 | 0.986 |
|  |  | Amphipod x Fish | -0.043 | 0.058 | -0.730 | 0.466 |
|  |  | Parasitism x Fish | -0.036 | 0.056 | -0.650 | 0.516 |
|  |  | Amphipod x Parasitism x Fish | 0.065 | 0.065 | 1.004 | 0.316 |
| *Baetis rhodani* | *a* | Intercept | 1.521 | 0.339 | 4.484 | **<0.001** |
|  |  | Amphipod | -0.728 | 0.381 | -1.911 | 0.056 |
|  |  | Parasitism | -0.168 | 0.460 | -0.365 | 0.715 |
|  |  | Fish | 0.201 | 0.522 | 0.384 | 0.701 |
|  |  | Amphipod x Parasitism | 0.792 | 0.702 | 1.128 | 0.259 |
|  |  | Amphipod x Fish | -0.152 | 0.573 | -0.265 | 0.791 |
|  |  | Parasitism x Fish | 0.129 | 0.677 | 0.1906 | 0.848 |
|  |  | Amphipod x Parasitism x Fish | 0.286 | 1.049 | 0.2728 | 0.785 |
|  | *h* | **Intercept** | 0.084 | 0.018 | 4.706 | **<0.001** |
|  |  | **Amphipod** | -0.054 | 0.026 | -2.074 | **0.038** |
|  |  | Parasitism | -0.007 | 0.025 | -0.2669 | 0.790 |
|  |  | Fish | 0.0143 | 0.024 | 0.5849 | 0.559 |
|  |  | **Amphipod x Parasitism** | 0.132 | 0.046 | 2.867 | **0.004** |
|  |  | Amphipod x Fish | -0.008 | 0.035 | -0.2279 | 0.820 |
|  |  | Parasitism x Fish | -0.051 | 0.031 | -1.627 | 0.104 |
|  |  | Amphipod x Parasitism x Fish | 0.017 | 0.057 | 0.2927 | 0.770 |

**
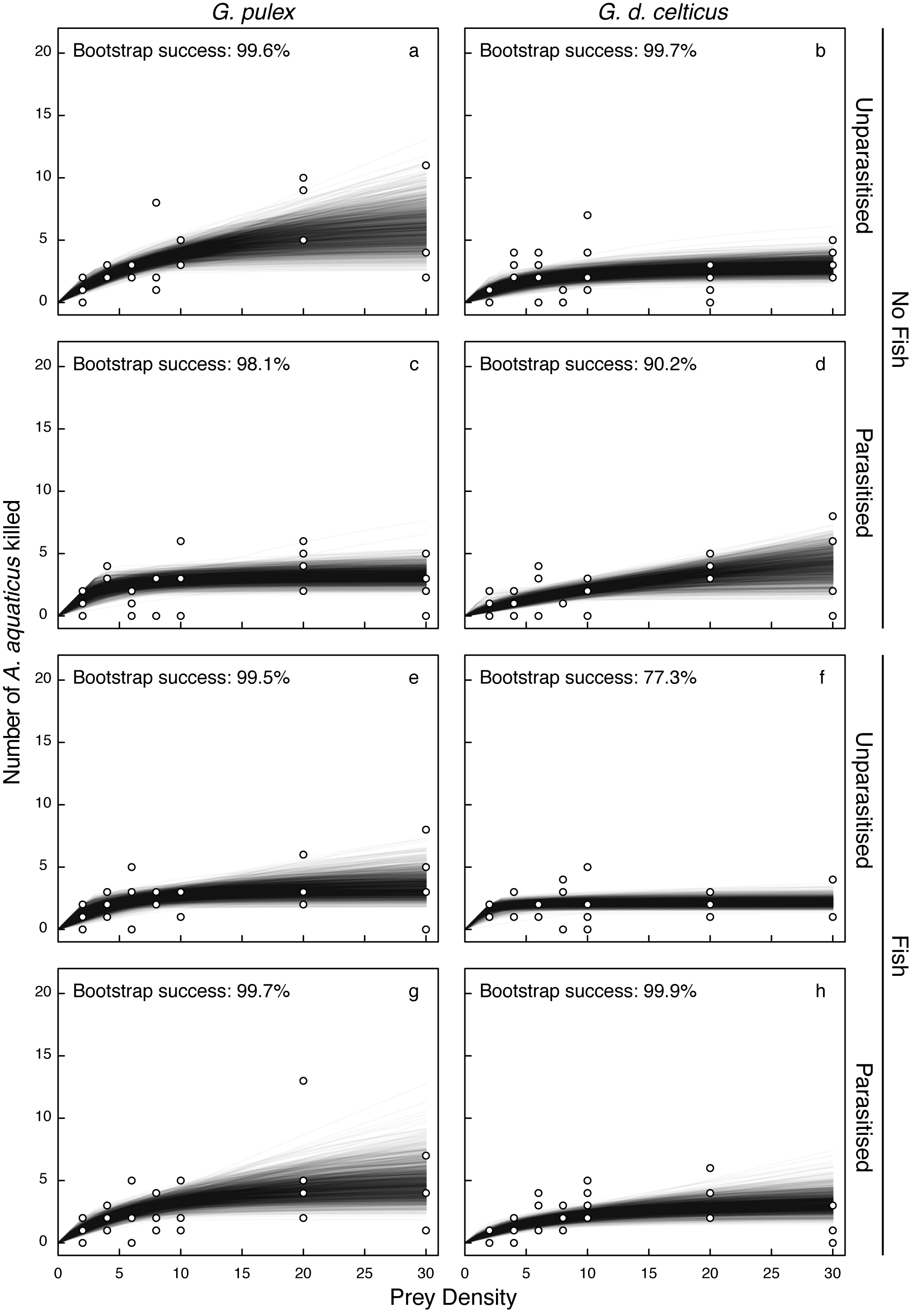
**

**Fig. S1.** The combined effects of parasitism and higher-order predator on the predatory functional responses of native *Gammarus duebeni celticus* (unparasitised or parasitised with *Pleistophora mulleri*) and invasive *Gammarus pulex* amphipods (unparasitised or parasitised with *Echinorhynchus truttae*) towards *Asellus aquaticus* prey. Bootstrap success rates are a measure of bootstrap stability and represent the percentage of the requested 1500 curves that fitted successfully with supplied starting values. Circles represent raw data points, lines represent bootstrapped functional responses (n = 1160 – 1498 depending on success rate).


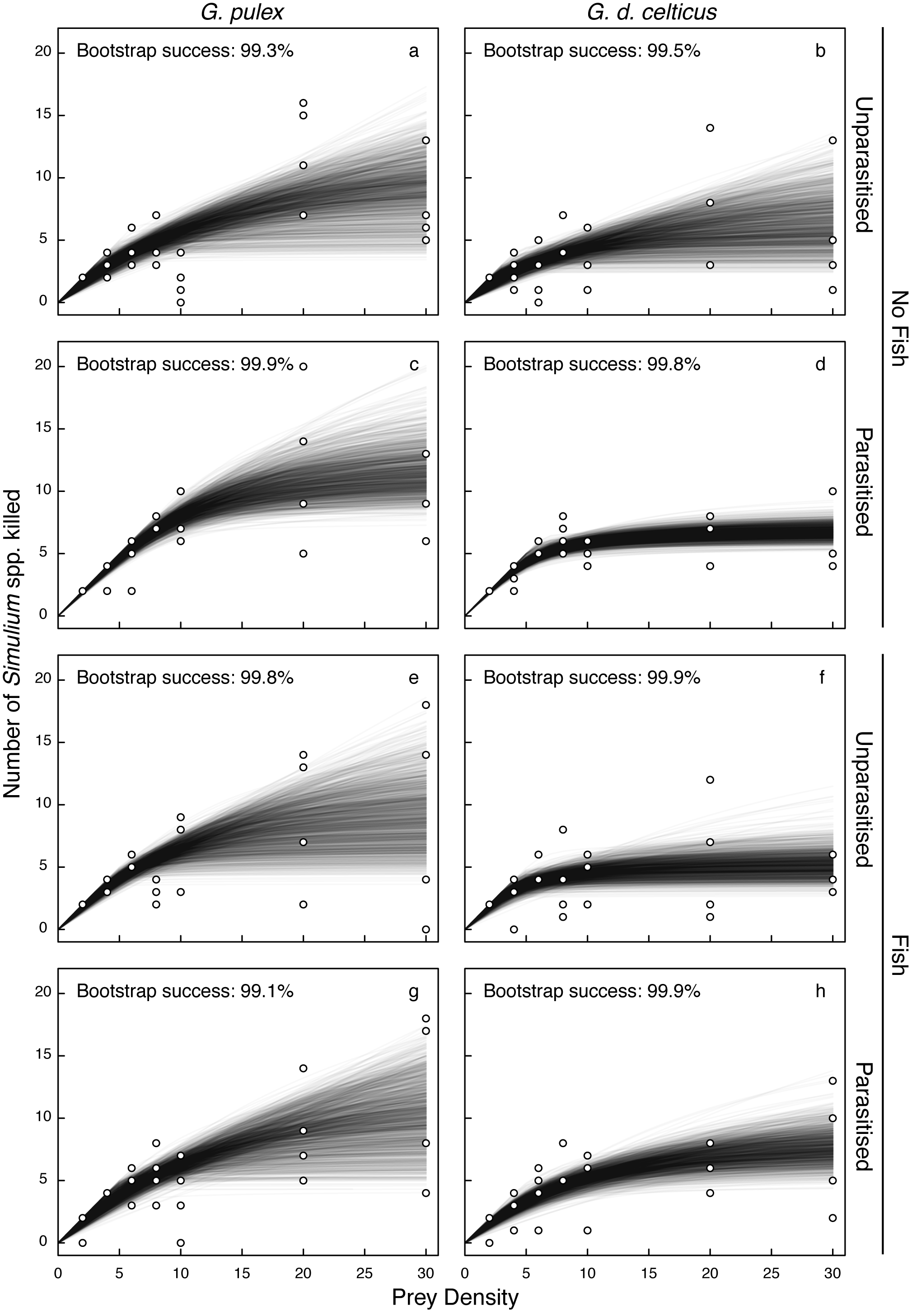


**Fig. S2.** The combined effects of parasitism and higher-order predator on the predatory functional responses of native *Gammarus duebeni celticus* (unparasitised or parasitised with *Pleistophora mulleri*) and invasive *Gammarus pulex* amphipods (unparasitised or parasitised with *Echinorhynchus truttae*) towards *Simulium* spp. prey. Bootstrap success rates are a measure of bootstrap stability and represent the percentage of the requested 1500 curves that fitted successfully with supplied starting values. Circles represent raw data points, lines represent bootstrapped functional responses (n = 1487 – 1499 depending on success rate).


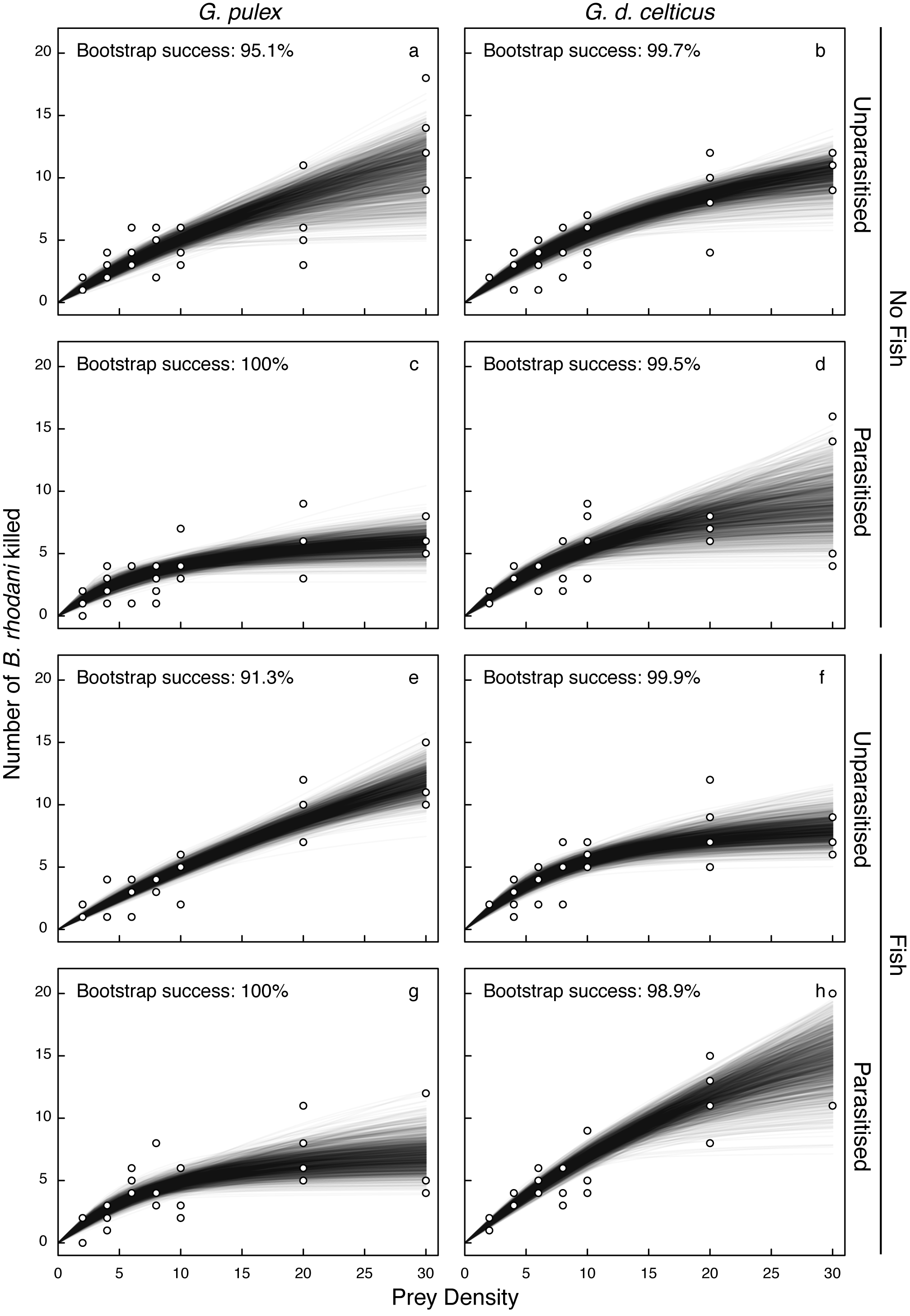


**Fig. S3.** The combined effects of parasitism and higher-order predator on the predatory functional responses of native *Gammarus duebeni celticus* (unparasitised or parasitised with *Pleistophora mulleri*) and invasive *Gammarus pulex* amphipods (unparasitised or parasitised with *Echinorhynchus truttae*) towards *Baetis rhodani* prey. Bootstrap success rates are a measure of bootstrap stability and represent the percentage of the requested 1500 curves that fitted successfully with supplied starting values. Circles represent raw data points, lines represent bootstrapped functional responses (n = 1369 – 1500 depending on success rate).
